# Genetic Heterogeneity of X-Linked Ichthyosis in the Republic of North Ossetia–Alania, Case Series Report

**DOI:** 10.3390/ijms24054515

**Published:** 2023-02-24

**Authors:** Tatyana A. Vasilyeva, Andrey V. Marakhonov, Inna S. Tebieva, Vitaly V. Kadyshev, Artem O. Borovikov, Zhanna G. Markova, Alyona L. Chukhrova, Evgeny K. Ginter, Sergey I. Kutsev, Rena A. Zinchenko

**Affiliations:** 1 Research Centre for Medical Genetics, 115522 Moscow, Russia; 2 Republican Children’s Clinical Hospital, 362003 Vladikavkaz, Russia; 3 N.A. Semashko National Research Institute of Public Health, 105064 Moscow, Russia

**Keywords:** X-linked ichthyosis, *STS* gene, gross chromosomal deletions, STR markers, North Ossetia–Alania

## Abstract

North Caucasus has always been a residence of a lot of different authentic ethnic groups speaking different languages and still living their traditional lifestyle. The diversity appeared to be reflected in the accumulation of different mutations causing common inherited disorders. X-linked ichthyosis represents the second most common form of genodermatoses after ichthyosis vulgaris. Eight patients from three unrelated families of different ethnic origin, Kumyk, Turkish Meskhetians, and Ossetian, with X-linked ichthyosis from the North Caucasian Republic of North Ossetia–Alania were examined. NGS technology was implied for searching for disease-causing variants in one of the index patients. Known pathogenic hemizygous deletion in the short arm of chromosome X encompassing the STS gene was defined in the Kumyk family. A further analysis allowed us to establish that likely the same deletion was a cause of ichthyosis in a family belonging to the Turkish Meskhetians ethnic group. In the Ossetian family, a likely pathogenic nucleotide substitution in the STS gene was defined; it segregated with the disease in the family. We molecularly confirmed XLI in eight patients from three examined families. Though in two families, Kumyk and Turkish Meskhetian, we revealed similar hemizygous deletions in the short arm of chromosome X, but their common origin was not likely. Forensic STR markers of the alleles carrying the deletion were defined to be different. However, here, common alleles haplotype is hard to track for a high local recombination rate. We supposed the deletion could arise as a de novo event in a recombination hot spot in the described and in other populations with a recurrent character. Defined here are the different molecular genetic causes of X-linked ichthyosis in families of different ethnic origins sharing the same residence place in the Republic of North Ossetia–Alania which could point to the existing reproductive barriers even inside close neighborhoods.

## 1. Introduction

Recessive X-linked ichthyosis (XLI) (OMIM #308100) represents one of the most common Mendelian forms of hereditary ichthyosis, the second most common one after ichthyosis vulgaris. Recessive X-linked ichthyosis is characterized by dark brown polygonal scales and general skin dryness. Skin involvement manifests soon after birth; histologically, it is described as compact hyperkeratosis and mild acanthosis with a normal granular layer. X-linked ichthyosis develops due to steroid-sulfatase deficiency resulting from the loss of function mutations, predominantly deletions of the *STS* gene (OMIM *300747) [1]. Steroid sulfatase hydrolyzes the cholesterol sulfate; the deficiency of its function leads to a lack of cholesterol, the accumulation of cholesterol sulfate, a deterioration of the normal process of exfoliation of the outer layer of the epidermis, and a violation of its barrier function [2,3]. The incidence of the XLI is 1 in 2500–6000 males [4,5]. Female carriers of the pathogenic allele usually have no manifestations of the disease since the locus responsible for the development of the disease does not undergo inactivation, unlike other regions of chromosome X [6]. Nonetheless, female carriers could suffer from severe corneal opacity [7]. Additionally, they could have a lower estrogen level due to placental steroid sulfatase deficiency during pregnancy [8]. The reason for that is in male fetuses with an STS function deficiency as almost all maternal estriol is converted in the placenta from precursors produced in the fetus. Mothers heterozygous for the *STS* gene mutation with female fetuses heterozygous for the mutation do not have placental steroid sulfatase deficiency. Women in the case of male fetuses with hemizygous deletion or other mutation of the *STS* gene could have placental steroid sulphatase deficiency, pregnancies at risk, and failed induction of labor [9].

During expedition, physicians of epidemiological studies experience diversity in clinical picture due to genetic and environmental factors. In addition, given the number of hereditary dermatoses and their genetic variability, as well as the necessity of a thorough understanding of disease, a precise genetic cause and epidemiological characteristics are essential.

In the course of expedition of the Research Centre for Medical Genetics (RCMG) to the Mozdoksky district of the Republic of North Ossetia–Alania (RNOA), eight patients from three unrelated families with hereditary ichthyosis were identified. The families belonged to the Kumyk, Turkish Meskhetians, and Ossetian ethnic groups. The study aimed to revel the genetic causes and genetic background of genodermatoses described in three families of different ethnic origins living nearby in one of the Northern Caucasus Republics, North Ossetia–Alania.

## 2. Results

For almost four decades, the staff of the Laboratory of Genetic Epidemiology of RCMG have been performing genetic and epidemiological studies of different regions of Russia. The protocol of comprehensive genetic research includes the examination of patients with suspected hereditary pathology by clinical geneticists, as well as other specialists, in order to verify the diagnosis, including the molecular genetic methods [10]. During an expedition to the Mozdoksky district of RNOA, eight patients from three unrelated families were diagnosed with XLI. The point prevalence of X-linked ichthyosis among the surveyed population of the Mozdoksky district of the Republic of North Ossetia–Alania was calculated. The prevalence of X-linked ichthyosis among the male population of the district was 1:5223 men (19.15 per 100,000 men), which corresponded to the Orphanet data (16.6 per 100,000) [5].

The families belonging to the Kumyk, Turkish Meskhetians, and Ossetian ethnic groups represented three peoples of the RNOA; seven out of them were available for clinical examination as well as for molecular diagnosis. A comparative clinical description of the patients identified is summarized in Table 1.

### 2.1. Clinical Description

#### 2.1.1. Family #102

**Figure 1:**
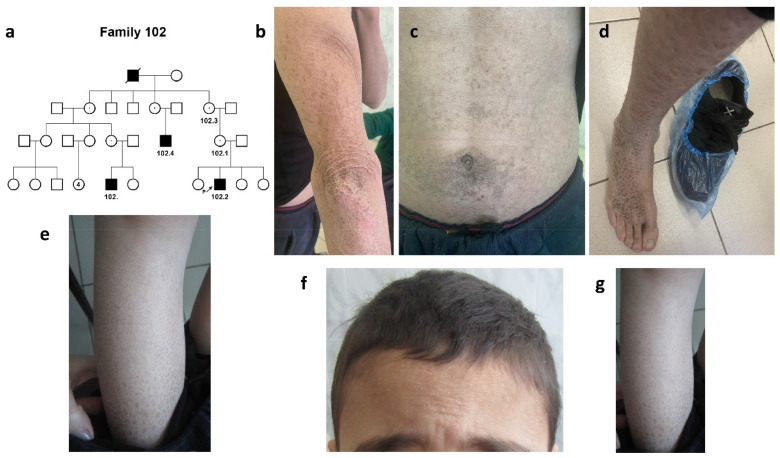
**Family #102.** (**a**) Pedigree #102. Four-generation pedigree #102 enclosed 8 nuclear families with 4 ichthyosis patients, #102.2, #102, #102.4, and their common great grandfather by their mothers; (**b**–**d**) photo of patient #102.4. Hyperkeratosis of the skin on the elbows (**b**), abdomen (**c**), dark-brown scaling on the leg and back of the foot (**d**); (**e**) photo of patient #102. Abundant “dirty” brown scales on the legs; (**f**,**g**) photo of patient #102.2. Dry scales on the forehead and hairy part of the head (**f**) and brown scaling on the legs (**g**).

A large four-generation pedigree #102 of Kumyk origin was represented by four ichthyosis patients, two cousins by their mothers, their uncle (maternal cousin), and their common great grandfather by their mothers (Figure 1a).

Both parents of the proband and three sisters were healthy.

In the family, severe X-linked ichthyosis was diagnosed. The disease in all patients manifested on the first days after their birth in the form of dry skin, infantile atopic dermatitis, and weakly expressed scales. Further, the scales (grayish, brown, or black) gradually affected almost the entire surface of the trunk, having a generalized nature of the lesion (Table 1).

The most severe clinical picture was noted in a 35-year-old patient #102.4 (Figure 1b–d).

Generalized dryness and hyperkeratosis of the skin were noted on the hands, legs, abdomen, and hairy part of the head. The scales were essentially brown or black in color. The severity of the course progressed with age. No other systems were involved.

In 7-year-old (#102) and 10-year-old (#102.2) patients, the disease was represented by the signs of “black” ichthyosis. There were abundant “dirty” brown scales on both arms, legs, the hairy part of the head, and the abdomen (Figure 1e–g, Table 1). No involvement of other organs and systems was detected in any of the patients, and no behavior disorder was noted.

#### 2.1.2. Family #47

**Figure 2:**
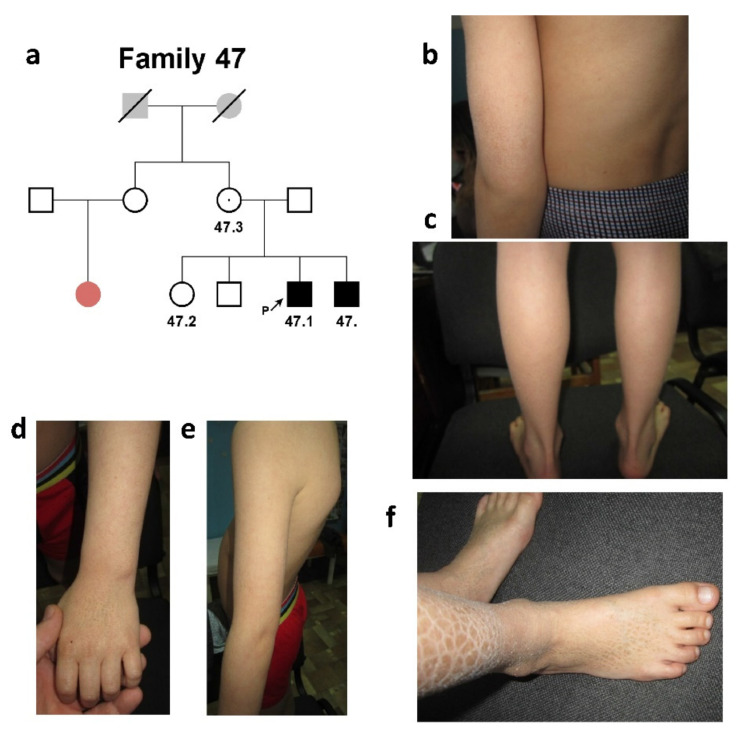
**Family #47.** (**a**) Three-generation pedigree #47 was presented by two affected siblings (#47 and #47.1). Probands’ female cousin was affected with palmoplantar hyperkeratosis (colored with pale red in the pedigree); (**b**,**c**) photo of patient #47.1. Small grayish-brown scales (2–5 mm in size) on the surface of the back and forearm (**b**), large dark scales (12–17 mm in size) on the lower back surface of the legs (**c**); (**d**–**f**) photo of patient #47. Grayish-brown scales of small size on the back and forearm (**d**,**e**) and larger scales on the legs (**f**).

The second family, #47 of Meskhetian Turkish origin, had two affected siblings. The proband’s sister, brother, and both his parents were healthy (Figure 2a).

The proband’s female cousin was affected with palmoplantar hyperkeratosis. Proband #47.1 was an 11-year-old male who had signs of “black” ichthyosis (Figure 2b,c; Table 1).

The affected area involved the back surface of the palm and forearm (small scales of the grayish-brown color of 2–5 mm in size), lower parts of the legs (large scales of the dark color of 12–17 mm in size), and the back surface of the foot (scales of the grayish-brown color of 2–5 mm in size). The disease manifested on the first days after birth in the form of infantile atopic dermatitis.

The proband’s sibs (Figure 2d–f) presented a similar clinical picture. Neither involvement of other systems was found in any of the brothers, nor were abnormalities of behavioral reactions noted.

#### 2.1.3. Family #143

**Figure 3:**
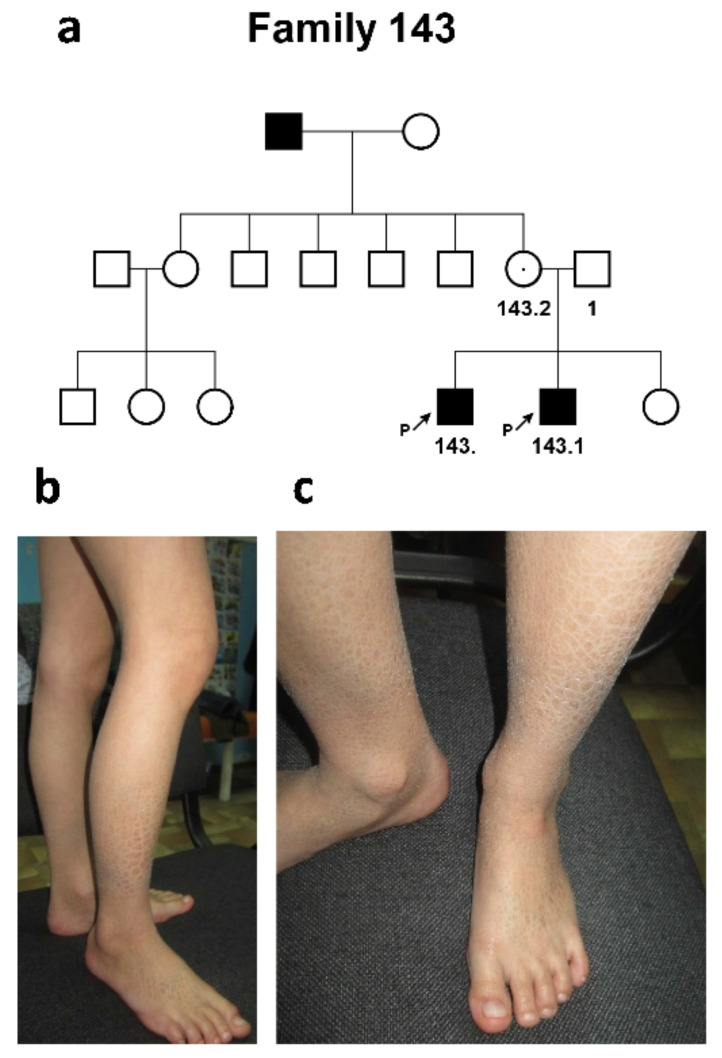
**Family #143.** (**a**) Three-generation pedigree #143 with 3 patients, 2 siblings (#143 and #143.1), and maternal father (who was not available for clinical examination and molecular diagnosis); (**b**,**c**) patient #143. Brown skin scales of various sizes, from small (2–3 mm) to larger (12–17 mm) on the leg.

The third, Ossetian, family #143 was represented by two affected siblings and the affected maternal father (according to the mother’s words). Both of the proband’s parents were healthy (Figure 3a).

Proband #143 was a 12-year-old male who had signs of a mild form of “black” ichthyosis, affecting only the shins and forearms characterized only by severe dryness of the skin. Generalized dryness of the skin and the formation of grayish-brown skin scales of various sizes—from small (2–3 mm) to larger (12–17 mm)—were noted (Figure 3b,c). The disease manifested from birth.

The proband’s sib, a 9-year-old male, had a similar clinical picture. No involvement of other systems was found in either the patient or the affected family members. No other systems were involved.

### 2.2. Molecular Genetic Diagnosis

WES in an index patient (#102.2) revealed a hemizygous deletion in the short arm of chromosome X seq[GRCh37] del(X)(p22.31)del chrX:(6302004_6966961)_(8434551_8496915)del (hg19) of 1.5–2.2 Mb in length encompassing the *PUDP*, *MIR4767*, *STS*, *VCX*, *PNPLA4*, *MIR651*, *VCX2*, and *VCX3B* genes. Hemizygous deletions of the region of similar sizes, including the *STS* gene, had been described in patients with XLI (OMIM# 308100) [National Center for Biotechnology Information. ClinVar; [VCV000446340.2], https://www.ncbi.nlm.nih.gov/clinvar/variation/VCV000446340.2 (accessed on 18 May 2022)]. The deletion affecting the entire *STS* gene is thought to have a strong loss-of-function effect, which was shown earlier [11].

**Figure 4:**
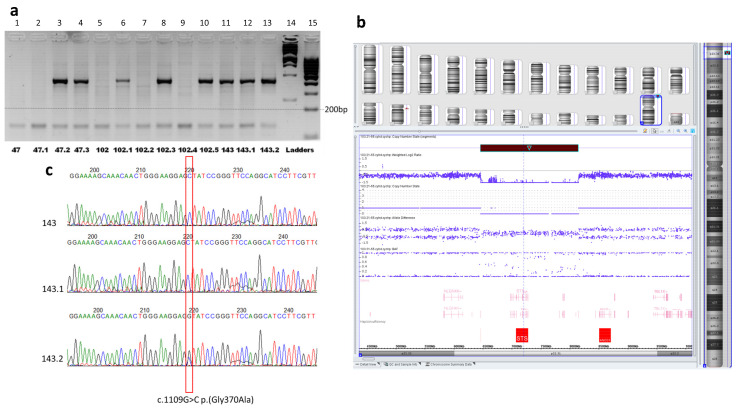
**Molecular genetic analysis of the families.** (**a**) Photo of the agarose gel electrophoresis of PCR products, two DNA fragments, from the deletion region (sequence of exon 7 of the STS gene, a fragment of 421 bp in size) and control fragment of the constitutive sequence (exon 4 of the PAX6 gene, a fragment of 150 bp in size). The internal fragment from the deletion (the *STS* gene exon 7) was absent in patients with XLI, ## 47, 47.1, 102, 102.2, and 102.4 (lanes 1, 2, 5, 7, 9). The two fragments one can see in lanes 3, 4, 6, 8, and 10 corresponding to healthy females from pedigrees #47 and #102, as well in the lanes 11, 12, and 13 corresponding to the members of family 143 without deletion. M12,1Kb DNA marker and M34, 100 bp DNA marker (SibEnzyme^®^, Moscow, Russia) were used as ladders (lanes 14 and 15). (**b**) CMA results for patient #102. The view of Chromosome Analysis Suite Software of CMA results focused on the Xp22.31 region demonstrating hemizygous deletion affected the entire *STS* gene with the adjacent sequences. (**c**) Results of Sanger sequencing of the *STS* gene exon 8 in family #143. Electropherograms demonstrate the variant NM_000351.7:c.1109G>C p.(Gly370Ala) in the hemizygous state in two male patients ## 143 and 143.1, and in heterozygous state in their healthy mother #143.2.

The deletion was further screened in the rest of the patients of the family #102 and later in two other families with XLI. In each of the examined individuals, two DNA fragments were amplified, one from the deleted region (exon 7 of the *STS* gene) and the second, a control fragment of a conservative sequence (exon 4 of the *PAX6* gene). In the patients from two out of the three examined families, the internal fragment from the deletion region corresponding to exon 7 of the *STS* gene was absent, indicating a similar deletion of the short arm of chromosome X (Figure 4a).

To determine more accurate boundaries of the revealed deletion, CMA analysis was performed in patient 102 (Figure 4b). The deletion position was defined arr[GRCh37] Xp22.31(6449559×1,6449753_8135644×0,8135645×1).

Further, to define possibly the same deletion-linked haplotype of the alleles at the STR markers loci, we analyzed three forensic STR markers, DXS10148, DXS10135, and DXS8378, localized at 1–2 Mb from the *STS* gene in the families #102 and #47. They showed that different alleles carried the deletion in the two families. Moreover, the high recombination rates of the X chromosomes in females also resulted in different alleles of the most distant for the deletion STR marker, DXS8378, in siblings within one family. The results of the genotyping are presented in Table 2.

In the third family, #143, the deletion was not revealed. Further searching for the *STS* gene pathogenic sequence variants was implied by Sanger sequencing. Two patients in this family were defined to have the variant chrX:g.7243407G>C (NM_000351.7(*STS*):c.1109G>C, p.(Gly370Ala)) in the hemizygous state, while their healthy mother was a heterozygous carrier of the variant (Figure 4c).

**Figure 5:**
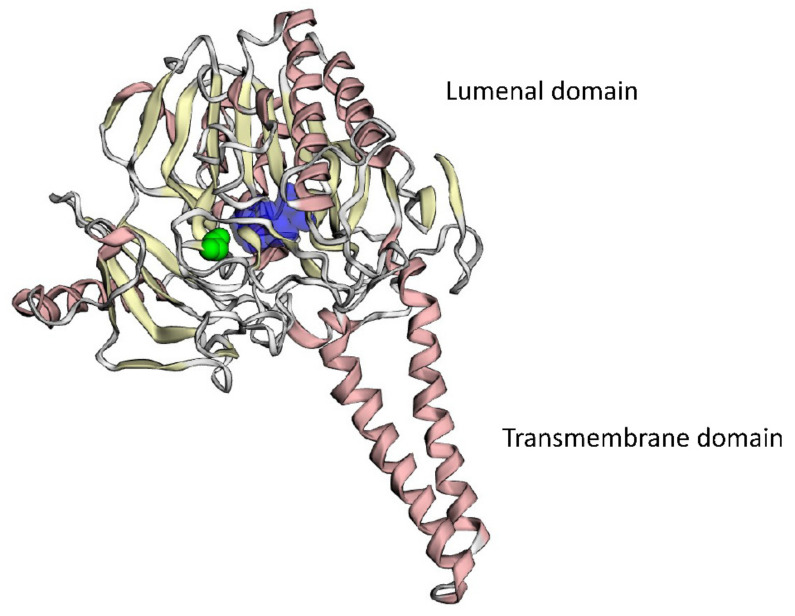
3D modeling of the mutant STS protein. Ca^2+^-binding cavity is colored dark blue, mutant amino-acid residue, p.G370A, is colored green.

The variant is absent in the GnomAD database [12]. This missense variant affects a very conservative position of the STS protein located nearby the 372-residue, of which the substitution of leads to the loss of steel–sulfatase activity [13]. PROVEAN, DEOGEN2, FATHMM-MKL, LRT, and M-CAP algorithms predict its deleterious consequences. The variant was reported once in ClinVar as likely pathogenic (variant ID: 450562). It co-segregates with the disease in the family. According to the ACMG/AMP criteria, the variant could be classified as likely pathogenic (PM2, PP3, PP1-S, PP5, PP4) [14]. The substitutions of the neighboring amino-acid residues, p.W372R and p.W372S, lead to a profound loss of the enzymatic activity of the STS protein. We performed bioinformatic analysis and 3D-modelling of the substitution, which suggested its deleterious effect on the protein function. The analysis revealed that G370-residue was in the internal region of the luminal domain of the enzyme and participated in the formation of the internal cavity for Ca^2+^-ions required for its hydrolase activity [15]. Glycine-to-alanine substitution introduced a hydrophobic aliphatic methyl group inside the tight hydrophilic space of the metal–ion binding site (Figure 5). Taken together, the analysis of the literature and the bioinformatic predictions along with the 3D modeling suggested that p.G370A substitution should have a significant effect on the enzymatic activity of the STS protein.

## 3. Discussion

During the expeditionary examination of patients with hereditary diseases in one of the eight districts of the RNOA, Mozdoksky district, situated in the North of the Republic, eight patients from three unrelated families with hereditary ichthyosis were referred to the doctors of the Research Centre for Medical Genetics for the diagnostics. The three families considered themselves to belong to Kumyk, Turkish Meskhetians, and Ossetian ethnic groups.

Kumyks represent the indigenous Caucasus population of the Kumyk plateau in RNOA and Dagestan. In the RNOA, the number of Kumyks is about ten thousand people. Kumyks speak the Kumyk language, belonging to the Kypchak group of Turkic languages.

Turkish Meskhetians are Turkic-speaking Muslims, a heterogeneous ethnic group of disputed origin. They come from the Meskheti region in southwestern Georgia and are considered either Turkized Georgians or related to Turks or Azerbaijanis. They speak the Kars dialect of the Turkish language.

The third, the Ossetian family, considered themselves to belong to Digors, a subethnic group of Ossetians, the indigenous population of the western part of North Ossetia. At the beginning of the 19th century, several Digor families moved to the territory of the modern Mozdoksky region. Digors speak the Digor dialect of the Ossetian language, belonging to the Iranian group of the Indo-European language family.

We observed clear and character clinical features of X-linked ichthyosis in all three families; however, clinical picture varied between and inside pedigrees. The disease in all patients manifested very early after birth as atopic dermatitis and later developed into generalized skin lesions (grayish, brown, or black scales) that affected the hairy part of the head, trunk, legs, and arms. No other organs or systems were involved in any of the patients. There were some peculiarities in the described cases of ichthyosis. We have found a severe clinical phenotype in at least two families with defined deletions. Usually, the milder phenotype is associated with deletions of the described size [16]. Intrafamilial clinical polymorphism was also observed in the families under investigation.

In two families, those of Turkish Meskhetian and Kumyk origin, presumably the same earlier reported pathogenic deletion of chromosome Xp22.31 encompassing the STS gene was revealed. Deletions of similar regions located between loci DXS1139 and DXF22S1 (mainly 1.9 Mb in length) were earlier identified in 19 out of 22 examined families of different origins from Israel and in 11 out of 12 examined families with XLI from Japan (OMIM # 308100) [17,18].

The deletion could have a recurrent character as that could occur as de novo in the same hot spot for the recombination chromosomal region between the flanking regions of the homologous low copy repeat sequences [19].

The founder effect was suggested. To assess the possible common origin of the revealed deletion in two Turkic families, three STR loci were analyzed. The length of the nearest to the deleted loci polymorphic markers in the probands from Turkish Meskhetians and Kumyks families was defined to be different (Table 2). That did not support the idea of the common origin of the allele with the deletion in the two Turkic families. However, it should be noted that even the nearest analyzed STR marker was located 1.1–1.3 Mb from the deletion. The distance could be too large to comprise a tightly linked haplotype with the deleted region because of a very high rate of recombination of X chromosome telomeres [20]. This high recombination rate within the region of interest is supported by the different alleles of the most distant STR locus DXS8378 in siblings even inside both families obviously due to recently happened recombination events. Thus, STR markers DXS10148, DXS10135, and DXS8378 could be hardly used to characterize ancestry haplotypes linked with the deleted region.

The cause of X-linked ichthyosis in the third family of Ossetian origin was a hemizygous novel single-nucleotide substitution in the exon 8 of the *STS* gene, NM_000351.7:c.1109G>C p.(Gly370Ala). According to ACMG recommendations assessment, bioinformatic analysis, and 3D modeling, this variant could be classified as probably pathogenic.

We could observe the clinical and genetic heterogeneity of XLI in the patients from one of the RNOA districts, the Mozdoksky district. The small size of the sample did not allow for correlating severity or peculiarities of the XLI clinical picture with its genetic cause.

Further analysis suggested searching for deletions break points, which are perhaps different in the two families. The significance of the conducted research was rather exploratory, though practical recommendations also could be made. Here, we emphasize again the inferred unacceptability of the characterization of the region by routinely using for haplotyping X chromosome STR markers for a very high local recombination rate. The second aspect of the practical significance and further work concerns genetic counseling of the women from the described Ossetian families who carry the deletion of the *STS* gene as in the case of the male fetus, they could have placental steroid sulphatase deficiency, pregnancies at risk, and the failed induction of labor.

## 4. Materials and Methods

### 4.1. Patients

The patients were examined during a field expedition to the RNOA, with the help of the local Ministry of Health Care and according to the Laboratory’s protocol of genetic epidemiological research designed to identify >3500 hereditary disorders [10]. Eight patients from three unrelated families (seven patients with accessible medical records and blood samples) from the Mozdoksky district of the RNOA were diagnosed with XLI. The diagnoses were made based on the available medical records, photographs, as well as skin, eyes, and a general clinical examination of the patients.

### 4.2. Informed Consent

Informed consent was obtained, as appropriate, from 7 subjects and/or their legal representatives included in the study. The study was conducted in accordance with the Declaration of Helsinki and approved by the Institutional Review Board of the Research Centre for Medical Genetics, Moscow, Russia (protocol no. 2017-4/1 dated 4 May 2017).

### 4.3. DNA Purification

Genomic DNA was isolated from peripheral blood leukocytes using the Wizard^®^ Genomic DNA Purification Kit (Promega, Madison, WI, USA) according to the method recommended by the manufacturer.

### 4.4. High-Throughput Sequencing

For the search for the causative variant in one of the index patients, whole exome sequencing (WES) was implied. WES was performed using a BGISEQ-500 instrument with an average on-target coverage of 146× with the MGIEasy Exome Capture V4 kit (BGI, Shenzhen, China) for library preparation (Genomed Ltd., Moscow, Russia). The analysis of the data was performed using an in-house software pipeline as described earlier [21]. That included the quality control of raw reads (FastQC tool v. 0.11.5) followed by read mapping to the hg19 human genome assembly (bwa mem v. 0.7.1), sorting of the alignments, and marking duplicates (Picard Toolkit v. 2.18.14). Base recalibration and variant calling were performed with GATK3.8. Variant annotation was conducted using an Annovar tool (v. 2018Apr16). Further filtering was performed by functional consequences and population frequencies according to the ACMG recommendations as well as clinical relevance determined by the Human Phenotype Ontology database [22].

### 4.5. Chromosomal Microarray Analysis

Further, more accurate deletion boundaries were defined by chromosomal microarray analysis (CMA). DNA samples were analyzed using chromosomal microarray analysis (CMA). The analysis was performed using the GeneChip™ 3000 system (Thermo Fisher Scientific, Waltham, MA, USA) according to the manufacturer’s protocol on a CytoScan HD microarray containing 2.67 million markers. Copy number variations (CNVs) analysis was performed using the Chromosome Analysis Suite software version 4.0 (Thermo Fisher Scientific Inc.).

### 4.6. AFLP-PCR Analysis

PCR was performed according to the standard protocols. Deletion screening in patients and their healthy relatives from three unrelated families was performed by multiplex PCR which amplified a fragment of the STS gene sequence (exon 7) and a control fragment of a constitutive nucleotide sequence (exon 4 of the *PAX6* gene), and their electrophoresis was carried out in a 3% agarose gel. Two pairs of primers were used, STS_7F 5′-CACCCACTGAGTAGGGCAAC, STS_7R 5′-TCTTGACACAAGATGTGGGCT and PAX6_4F 5′-TGCAGCTGCCCGAGGATTAA, PAX6_4R 5′-CCGAAGTCCCAGAAAGACCA [23].

### 4.7. Polymerase Chain Reaction (PCR), Sanger Sequencing

Sanger sequencing was implied for searching for the STS gene pathogenic variants in the third family without *STS* gene deletion. PCR was performed according to the standard protocols. Two pairs of primers for exons 7 and 8, which were hot spots for mutations in the STS gene, were designed, forward and reverse primers for ехоn 7, STS_7F, and STS_7R were the same (see above), and for ехоn 8, they were as follows STS_8F 5′-AGATGCCATACAGTGCTGACC, STS_8R 5′-TTATGCAGGCAAACCAGAGCA. The fragments were purified using the Concert kit using NucleoFast 96 PCR plates and then analyzed by direct sequencing using an ABI PRISM 3100 DNA sequencer. The variant was named according to the reference transcript variant NM_000351.7 of the *STS* gene.

### 4.8. Amplified Fragment Length Polymorphism of STR Markers Loci Analysis

Alleles of three highly polymorphic STR markers, DXS10148, DXS10135, and DXS8378, localized at 1–2 Mb from the *STS* gene, were defined in the patients [24,25].

The loci were amplified with the Investigator Argus X-12 kit (QIAGEN, Hilden, Germany) according to the manufacturer’s instructions. Typing was done on the ABI PRISM 3130 Genetic Analyzer using GeneScan and Genotyper 3.7 software (Applied Biosystems, Waltham, MA, USA) in comparison to sequenced allelic ladders and cell lines of a known genotype (K562, 9947A, XX74).

For the interpolation of hg19 physical positions into the sex-averaged map positions in female Kosambi cM, Rutgers Maps v. 3 were used [26].

### 4.9. 3D Modeling of Mutant Protein

MuPIT interactive online tool was used to model the 3D structure of the STS protein [27]. The homology model of STS (Structure ID: NP_000342.2_1) was used for the prediction.

### 4.10. Calculation of the Point Prevalence Rate of the Disease

To calculate the prevalence rate, the male population was considered to be 46.3% of the population [https://infotables.ru/statistika/31-rossijskaya-federatsiya/200-sootnoshenie-chislennosti-muzhchin-i-zhenshchin-rossii-v-2010-i-2002-godakh-tablitsa, accessed on 5 September 2022]. The surveyed population of Mozdoksky district identified 90,244 surveyed people (41,783 were men).

## 5. Conclusions

We revealed eight patients with XLI from three examined families which were molecularly confirmed. The causes of XLI were the intragenic variant in the *STS* gene defined in the Ossetian family, and chromosome deletions encompassing the *STS* gene revealed in two other families, the Kumyk and Turkish Meskhetian ones. The founder effect could be suggested if we had several cases from the same ethnic group, which is not the case. Despite the fact that here the founder effect is less possible, the common alleles (founder) haplotype is hard to track for a high local recombination rate. When testing the origin of the alleles carrying the deletions, different allele haplotypes in two unrelated families were revealed. Defined deletion seemed to have a recurrent character and was often detected in worldwide populations arising at chromosome X22p. These circumstances may indicate the genetic heterogeneity of ichthyosis and its non-trivial molecular diagnosis, as well as the existence of marriage assortativeness and existing reproductive barriers even inside one region in the Northern Caucasus where numerous different people are living in the closest neighborhood.

## Figures and Tables

**Table 1:** Patients’ presentation, clinical picture, and genotype of seven patients with XLI from RNOA.

Patient	#102	#102.2	#102.4	#47	#47.1	#143	#143.1
Genotype at chrXp22.31 region or NM_000351.7 (*STS*)	g.[chrXp22.31del];[0]	g.[chrXp22.31del];[0]	g.[chrXp22.31del];[0]	g.[chrXp22.31del];[0]	g.[chrXp22.31del]; [0]	c.[1109G>C];[0]	c.[1109G>C];[0]
Ancestry	Kumyks	Kumyks	Kumyks	Turkish Meskhetians	Turkish Meskhetians	Ossetians	Ossetians
Age/gender	7 y.o./male	10 y.o./male	35 y.o./male	11 y.o./male	7 y.o./male	12 y.o./male	9 y.o./male
Dry skin, infantile atopic dermatitis, and mild scaling appeared in the first few weeks of life	+	+	n/d	+	+	+	+
Tightly adherent, brown scales at the surface of the extremities	+	+	+	+	+	+	+
Scales at the surface of the trunk and neck	+	+	+	+	none	none	none
Scales on the forehead	+	+	+	none	none	none	none
Scales on the hairy part of the head	+	+	+	none	none	none	none

**Table 2:** Genotypes by the deletion Xp22.31del and three STR markers were revealed in patients from families #47 and #102 and STR markers’ chromosomal location.

Loci Location and Patients’ ID	delXp22.31 (Encompassing the *STS* Gene)	DXS10148	DXS10135	DXS8378	Ethnic Origin
GenBank accession numbers		KC662330.1	MT607405.1	MT132938.1	
Chromosomal location (hg19) (Distance from a deletion in Mb)	chrX:6449753_8135644 (0 Mb)	chrX:9238969_9239205 (1.1 Mb)	chrX:9306118_9306616 (1.2 Mb)	chrX:9370150_9370429 (1.3 Mb)	
Female map position in Kosambi cM (Distance from deletion)	16.2–18.4 cM (0 cM)	20.2 cM (1.79 cM)	20.4 cM (1.94 cM)	20.6 cM (2.15 cM)	
#102	[chrXp22.31del];[0]	18	23.1	10	Kumyks
#102.2	[chrXp22.31del];[0]	18	23.1	9	Kumyks
#102.4	[chrXp22.31del];[0]	18	23.1	10	Kumyks
#47	[chrXp22.31del];[0]	27.1	20	9	Turkish Meskhetians
#47.1	[chrXp22.31del];[0]	27.1	20	10	Turkish Meskhetians

## Data Availability

The datasets used and/or analyzed during the current study are available from the corresponding author upon reasonable request.
